# Association between beta-blocker utilization and heart failure mortality in the peritoneal dialysis population: a cohort study

**DOI:** 10.1093/ckj/sfae022

**Published:** 2024-02-09

**Authors:** Meizhu Gao, Han Chen, Fang Cao, Li Zhang, Yiping Ruan, Weihua Liu, Fuyuan Hong, Jiewei Luo, Miao Lin

**Affiliations:** Department of Nephrology, Shengli Clinical Medical College of Fujian Medical University, Fujian Provincial Hospital, Fuzhou, China; The Third Department of Critical Care Medicine, Shengli Clinical Medical College of Fujian Medical University, Fujian Provincial Hospital, Fuzhou, China; Department of Nephrology, Shengli Clinical Medical College of Fujian Medical University, Fujian Provincial Hospital, Fuzhou, China; Department of Nursing, Shengli Clinical Medical College of Fujian Medical University, Fujian Provincial Hospital, Fuzhou, China; Department of Nephrology, Shengli Clinical Medical College of Fujian Medical University, Fujian Provincial Hospital, Fuzhou, China; Department of Nephrology, Shengli Clinical Medical College of Fujian Medical University, Fujian Provincial Hospital, Fuzhou, China; Department of Nephrology, Shengli Clinical Medical College of Fujian Medical University, Fujian Provincial Hospital, Fuzhou, China; Department of Nephrology, Shengli Clinical Medical College of Fujian Medical University, Fujian Provincial Hospital, Fuzhou, China; Department of Traditional Chinese Medicine, Shengli Clinical Medical College of Fujian Medical University, Fujian Provincial Hospital, Fuzhou, China; Department of Nephrology, Shengli Clinical Medical College of Fujian Medical University, Fujian Provincial Hospital, Fuzhou, China

**Keywords:** CAPD, dialysis, heart failure, hypertension, peritoneal dialysis

## Abstract

**Background:**

The prognostic significance of beta(β)-blocker therapy in patients at end-stage renal disease, specifically those receiving peritoneal dialysis (PD) and presenting with heart failure, remains inadequately elucidated due to limited research conducted thus far.

**Methods:**

A retrospective analysis was performed on a cohort comprising 608 patients receiving PD between September 2007 and March 2019, with a subsequent follow-up period extending until December 2020. Cox regression and propensity score matching weighted analysis was used to model adjusted hazard ratios for β-blocker use with heart failure-related mortality. Competing risk analysis and subgroup analysis were carried out to further elucidate the correlation.

**Results:**

β-blockers were prescribed for 56.1% of the peritoneal dialysis patients. Heart failure occurred in 43.4% of the total population and 15.5% of deaths were due to heart failure. The prescription of β-blockers was associated with a 43% lower adjusted hazard ratio (HR) for heart failure death within the cohort (95% confidence interval [CI] = 0.36–0.89; *P* = 0.013). Even after accounting for competing risk events, patients in the group using β-blockers demonstrated a significantly lower cumulative risk of heart failure-related mortality compared to those not using β-blockers (*P* = 0.007). This protective effect of β-blockers was also observed in subgroup analyses. Conversely, β-blocker use had no statistically significant associations with all-cause mortality.

**Conclusion:**

The use of β-blockers was associated with a reduced risk of heart failure-related mortality in the PD population. Future randomized clinical trials are warranted to confirm the beneficial effect of β-blockers in the context of PD.

KEY LEARNING POINTS **What was known**:

- Heart failure with preserved ejection fraction (HFpEF) is a prevalent condition among patients undergoing peritoneal dialysis.

**This study adds**:

- The use of β-blockers was associated with a reduced risk of heart failure-related mortality in the peritoneal dialysis population.

**Potential impact**:

- Future randomized clinical trials are warranted to confirm the beneficial effect of β-blockers in the context of peritoneal dialysis.

## INTRODUCTION

Chronic kidney disease (CKD), recognized as one of the most prevalent comorbidities, has been linked to severe heart failure (HF) and unfavorable cardiovascular outcome [1, 2]. Peritoneal dialysis (PD) is utilized as a treatment modality for approximately 11% of individuals worldwide suffering from kidney failure [3]. Among PD patients, the prevalence of heart failure is estimated to be around 35% or potentially higher [4]. This prominent complication, frequently observed in long-term PD cases, is associated with an increased risk of adverse clinical outcomes [5].

Beta(β)-blockers are cornerstone agents in the pharmacological management of heart failure patients [6]. They are also recommended as class I therapies for individuals with heart failure and reduced ejection fraction (HFrEF) according to guideline [7, 8]. Previous studies have demonstrated the favorable effects of β-blockers in reducing the risk of all-cause mortality, as well as combined endpoints of all-cause/cardiovascular death or HF hospitalization, in patients with HFrEF [9–13]. Additionally, evidence suggests that β-blockers have been shown to improve outcomes in patients with HFrEF in all stages of CKD [14–17]. A meta-analysis conducted in 2020 reveals that β-blocker therapy is associated with a reduction in the risk of all-cause mortality among patients with heart failure and chronic kidney disease (RR 0.69, 95% CI 0.60–0.79) [18]. Furthermore, β-blocker therapy has been associated with improved mortality rates in patients with dilated cardiomyopathy who undergo hemodialysis [19]. Several observational studies have demonstrated the advantageous effects of β-blocker use on left ventricular function and all-cause mortality among incident hemodialysis patients with HF [20–23]. However, studies specifically evaluating the risk of heart failure-related mortality in PD patients receiving β-blockers are lacking. The BLOCADE study was a randomized controlled trial that attempted to investigate the potential effect of β-blockers on cardiovascular mortality in PD patients, a definitive answer to this research question remains elusive due to insufficient sample size recruitment [24].

All of these studies suggest that there is an ongoing concern among researchers as to whether there may be a cardiovascular benefit from the use of β-blockers in the peritoneal dialysis population. Given this background, this cohort study was designed to further investigate the benefit of β-blockers in the PD population, primarily the effect on heart failure mortality.

## MATERIALS AND METHODS

### Study design and data source

A retrospective study was conducted to investigate patients with end-stage renal disease (ESRD) who initiated peritoneal dialysis at Fujian Provincial Hospital between September 2007 and March 2019. The study protocol complies with the Declaration of Helsinki and has full approval from the local Clinical Research Ethics Committee.

Baseline and follow-up data of the patients were extracted from their medical records at the time of initial PD treatment. Baseline information encompassed variables such as sex, age, body mass index (BMI), blood pressure, medication usage, urine volume, presence of diabetes and cardiovascular diseases, and history of HF. Additionally, various laboratory tests were collected, including measurements of serum calcium, phosphorus, albumin, hemoglobin, parathyroid hormone, N-terminal pro-B-type natriuretic peptide (NT-BNP), estimated glomerular filtration rate (eGFR), and Charlson comorbidities index (CCI). The CCI was calculated for each patient as a weighted total of their comorbid conditions, serving as a means to stratify patients and control for potential confounding effects on overall survival [25]. During follow-up, echocardiographic parameters, urine volume, ultrafiltration volume, total weekly urea clearance index (KT/V) and medication use were collected after 3 months of dialysis initiation. KT/V was calculated using the formula recommended in the K/DOQI guideline [26]. Echocardiographic parameters including left ventricular ejection fraction (LVEF), left ventricular end-diastolic volume index (LVEDVI), and left ventricular mass index (LVMI) were also documented. LVMI and LVEDVI were calculated from the corresponding echocardiographic measurements and body surface area according to the formulas used in previous literature [27]. Follow-up of all patients continued until death, discontinuation of PD, or 31 December 2020.

### Study population

**Figure 1:**
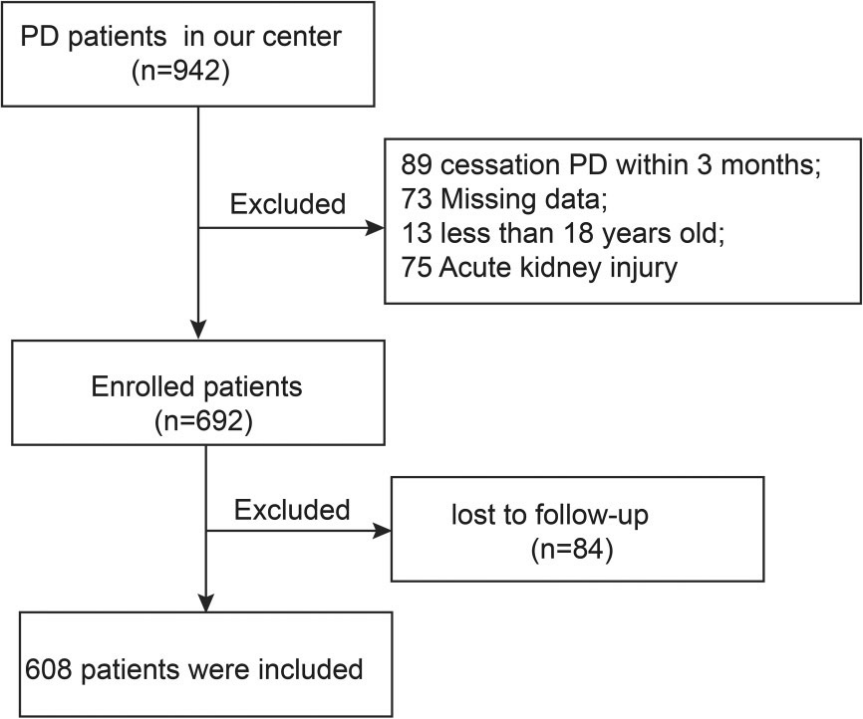
Flow chart of the study population.

In order to be eligible for participation in this study, individuals who were at least 18 years old and had been undergoing PD for a minimum of 3 months were considered. Additionally, it was necessary for patients to have available medication information. Exclusion criteria involved patients with acute kidney injury, PD duration less than 3 months, or insufficient follow-up data (missing medication information data). The flow chart for recruitment is illustrated in Fig. 1.

### Study exposure and outcome

Time zero for each patient was the initial PD treatment, with β-blockers (e.g. carvedilol, bisoprolol, or metoprolol) use defined at that time and subsequent outcomes of HF-related death and all-cause death. The primary clinical outcome of interest in this study was heart failure-related mortality as determined by hospitalization records. Secondary outcome was all-cause mortality as determined using the peritoneal dialysis center's data registry system.

HF was defined as clearly documented episodes requiring hospitalization. The diagnosis of HF was clinically established by the attending physician, guided by the following criteria [5, 28]: (i) manifestation of HF signs and symptoms, such as dyspnea, orthopnea, elevated jugular venous pressure, and a laterally displaced apical impulse, resulting from structural and/or functional abnormalities of the heart; (ii) identification of pulmonary venous congestion or interstitial edema through radiographic examinations; and (iii) observed resolution of symptoms, signs, and radiographic changes following hypertonic PD exchange. According to the 2016 European Society of Cardiology guidelines for the management of HF, subsets of HF include reduced ejection fraction, <40% (HFrEF); mid-range ejection fraction, 40% to 49% (HFmrEF); and preserved ejection fraction, ≥50% (HFpEF) [7]. Previous HF in this study pertains to individuals who have encountered HF episodes that were clearly documented to required hospitalization before the initiation of PD treatment. Recurrent HF refers to the occurrence of a previous history of HF followed by subsequent episodes of HF during the follow-up period of PD. The above information was obtained from the medical record system of Fujian Provincial Hospital and the Peritoneal Dialysis Registry Database. These databases maintain thorough records of all hospitalization episodes.

### Statistical analysis

Categorical variables were represented using numbers and percentages, while quantitative variables were presented as either median (25th to 75th percentile) or mean ± standard deviation (SD). Statistical differences in percentages, medians, and means across groups were assessed using the chi-square test, Kruskal–Wallis H test, and one-way analysis of variance (ANOVA), respectively. To analyse heart failure mortality, the Kaplan–Meier method was employed, and a log-rank test was conducted to compare the survival curves. Cox regression analysis was utilized to investigate the association between β-blocker prescription at the initiation of peritoneal dialysis and heart failure mortality. The study included results from two progressively adjusted model to reduce confounding. Model 1 comprised covariates with standardized differences >10%, and *P* < 0.1 in univariate regression analysis. Model 2 integrated model 1 along with covariates that may have clinical relevance or have been identified in previous studies as potential contributors to HF.

To assess the robustness of our findings, several sensitivity analyses were performed. First, to minimize the potential bias of treatment allocation and confounding, we performed 1:1 propensity score matching (PSM) to estimate the association between β-blocker use and outcomes by Cox regression. A 1:1 nearest neighbor matching algorithm with a caliper width of 0.01 was used. Standardized mean difference (SMD) was used to assess the magnitude of PSM. The estimated propensity scores were used as weights to generate weighted cohorts using an inverse probability of treatment weighting (IPTW) model. Second, we applied Fine and Gray's sub-distribution hazards regression model to examine the possible influence of competing events on the association between β-blockers and HF-related mortality. Finally, we performed subgroup analyses according to age group, sex, diabetes, coronary heart disease, hypertension, and smoking using Cox regression models. Additionally, the likelihood ratio test was carried out to explore potential interactions among subgroups.

The multiple imputation method was applied to handle missing data of covariates using Fully Conditional Specification (FCS) implemented by MICE algorithm as described by Van Buuren and Groothuis-Oudshoorn [29].

All statistical analyses were performed using Free Statistics software version 1.8 and the R software packages (http://www.R-project.org, The R Foundation). A two-sided *P*-value below 0.05 was considered statistically significant in all tests.

## RESULTS

### Baseline parameters of study population

Following the enrollment process, a total of 608 patients (63.5% male, 36.5% female) were enrolled in the present investigation, of which 35 had missing BMI data, and 10 had missing NT-BNP data.

Baseline characteristics of all participants, stratified by whether they have HF history based on β-blockers usage, are presented in Table 1. Among the 608 individuals undergoing peritoneal dialysis, 136 (22.4%) subjects have previous HF history while 472 (77.6%) patients have not. In the total group, 341 subjects (56.1%) received β-blocker therapy while the remaining 267 patients (43.9%) did not. The mean age was found to be 49.8 ± 15.7 years for β-blocker users, whereas non-users had a slightly higher mean age of 56.2 ± 15.9 years. A noteworthy prevalence of hypertension, diabetes, and coronary heart disease was observed, with rates recorded at 53.5%, 22.6%, and 9.7%, respectively. It is worth emphasizing that the total group receiving β-blockers exhibited elevated levels of diastolic blood pressure. As a crude proxy data for volume status, NT-BNP did not differ between patients on β-blockers and those not on β-blockers.

**Table 1:** Baseline characteristics of the study population according to β-blocker medication.

	Without previous HF		With previous HF	
	β-blocker non-use	β-blocker use	β-blocker non-use	β-blocker use
Variables	(*n* = 206)	(*n* = 266)	(*n* = 61)	(*n* = 75)
Demographic factors				
Age, y	53.8 ± 15.8	47.9 ± 15.2^a^	64.2 ± 13.8	56.2 ± 15.7^a^
BMI, kg/m^2^	22.1 ± 2.9	22.8 ± 3.8^a^	23.2 ± 3.4	23.5 ± 4.5
SBP, mm Hg	148.1 ± 22.3	151.4 ± 21.3	141.6 ± 21.7	150.5 ± 19.1^a^
DBP, mm Hg	83.4 ± 14.2	85.4 ± 14.6	79.0 ± 13.4	81.5 ± 13.5
Male, *n* (%)	124 (60.2)	168 (63.2)	43 (70.5)	51 (68)
Smoking, *n* (%)	42 (20.4)	48 (18.0)	18 (29.5)	22 (29.3)
Hypertension, *n* (%)	97 (47.1)	133 (50.0)	44 (72.1)	51 (68.0)
Diabetes, *n* (%)	41 (19.9)	63 (23.7)	23 (37.7)	34 (45.3)
CHD, *n* (%)	2 (1.0)	1 (0.4)	25 (41.0)	31 (41.3)
CCI	4.0 (3.0, 6.0)	3.5 (2.0, 6.0)^b^	8.0 (5.0, 10.0)	7.0 (5.0, 9.0)
Daily UV, dL	9.0 (4.7, 12.0)	7.9 (3.2, 11.0)	7.5 (3.0, 10.0)	7.0 (3.0, 12.0)
Laboratory exam				
Hemoglobin, g/L	86.8 ± 21.5	86.9 ± 19.6	83.9 ± 17.8	86.3 ± 18.9
Serum albumin, g/L	30.4 ± 7.9	31.3 ± 8.8	29.4 ± 5.1	28.9 ± 6.2
Ca × P, mg/dL	46.1 ± 16.7	50.7 ± 17.5^a^	45.6 ± 15.6	47.4 ± 15.6
eGFR,ml/min/1.73 m^2^	4.7 (3.8, 6.6)	4.6 (3.4, 6.0)	5.4 (4.0, 8.6)	5.4 (4.3, 7.4)
PTH, pmol/L	21.7 (10.3, 39.9)	22.0 (11.4, 37.0)	22.2 (12.3, 32.3)	18.0 (10.0, 32.1)
NT-BNP, pg/ml	8993 (2020, 23 995)	11 248 (3402, 35 000)	8831 (1758, 28 849)	9825(1850, 34 639)
Medications, *n* (%)				
CCB	163 (79.1)	249 (93.6)	45 (73.8)	71 (94.7)
Statin	65 (31.6)	79 (29.7)	23 (37.7)	38 (50.7)
ACEI or ARB	52 (25.2)	126 (47.4)	23 (37.7)	39 (52.0)
Antiplatelet agent	47 (22.8)	50 (18.8)	28 (45.9)	38 (50.7)
Erythropoietin	182 (88.3)	238 (89.5)	52 (85.2)	65 (86.7)

ACEI: angiotensin-converting enzyme inhibitor; ARB: angiotensin II receptor blocker; BMI: body mass index; Ca × P: Calcium-phosphorus product; CCB: calcium channel blockers; CCI: Charlson comorbidities index; CHD: coronary heart disease; DBP: diastolic blood pressure; NT-BNP: N-terminal pro-B-type natriuretic peptide; PTH: parathyroid hormone; SBP: systolic blood pressure; UV: urine volume.

^a^ *P*<0.01 by 2-tailed *t* test vs patients without β-blocker therapy.

^b^ *P*<0.01 by chi-square test vs patients without β-blocker therapy.

### Outcomes and follow-up data

During a median follow-up duration of 36.5 months (interquartile range [IQR]: 14–63 months), a total of 200 patients (32.9%) experienced mortality, with 94 of those deaths attributed to HF. Notably, HF episodes occurred in 264 patients (43.4%) within the cohort, with 170 classified as non-fatal incidents. Specifically, among the HF patients, 152 individuals (57.6%) experienced *de novo* HF, while 112 individuals (42.4%) had recurrent HF. The recurrence rate of HF was 82.4% in 136 patients with a history of HF.

Of the 264 patients who developed HF, 29 had missing follow-up data for echocardiographic parameters. According to established guidelines [7], in our study, LVMI (>95 g/m^2^ for female or >115 g/m^2^ for male), LVEDVI (>86 ml/m^2^), and LVEF were employed as proxy of metrics for assessing left ventricular hypertrophy and dysfunction. Notably, 72.5% of patients with HF exhibited left ventricular (LV) hypertrophy, 13.1% had LV diastolic insufficiency, and 10.6% had LV systolic insufficiency. It is noteworthy that 89.4% of patients with HF presented with LVEF ≥50%, which was defined as HFpEF. In addition, at a median follow-up of 20 months of incident heart failure, 70.5% of patients demonstrated urine output exceeding 400 mL, 29.2% had a urine output ranging between 100-400 mL, and only 0.4% were anuric. In terms of ultrafiltration volume of PD, 64.4% of patients exhibited a urine volume exceeding 500 ml. The above follow-up data are presented in Table 2.

**Table 2:** Outcome events and follow-up data stratified by HF and β-blocker use.

	Total HF	β-blocker non-use	β-blocker use	
Variables	(*n* = 264)	(*n* = 111)	(*n* = 153)	*P* value
Follow-up time	20.0 (11.0, 37.0)	16.0 (9.5, 27.0)	24.0 (12.0, 43.0)	
Death, *n* (%)	94 (35.6)	53 (47.7)	41 (26.8)	<0.001
De novo HF, *n* (%)	152 (57.6)	58 (52.3)	94 (61.4)	0.136
Recurrent HF, *n* (%)	112 (42.4)	53 (47.7)	59 (38.6)	0.136
LVEF, *n* (%)				0.913
<50%	25 (10.6)	11 (10.9)	14 (10.4)	
≥50%	210 (89.4)	90 (89.1)	120 (89.6)	
LVEDVI, *n* (%)^a^	30 (13.1)	14 (14.7)	16 (11.9)	0.537
LVMI, *n* (%)^b^	169 (72.5)	69 (71.1)	100 (73.5)	0.790
PD exchanges, (L)	6.2 ± 2.1	6.2 ± 2.2	6.3 ± 2.1	0.857
UFV, *n* (%)				0.975
<500 ml	94 (35.6)	39 (35.1)	55 (35.9)	
500–1000 ml	86 (32.6)	37 (33.3)	49 (32)	
>1000 ml	84 (31.8)	35 (31.5)	49 (32)	
UV, *n* (%)				0.492
<100 ml	1 ( 0.4)	0 (0)	1 (0.7)	
100–400 ml	77 (29.2)	29 (26.1)	48 (31.4)	
>400 ml	186 (70.5)	82 (73.9)	104 (68)	
Total weekly Kt/V	1.8 ± 0.6	1.8 ± 0.6	1.8 ± 0.5	0.947

LVEF: left ventricular ejection fraction; LVEDVI: left ventricular end-diastolic volume index; LVMI: left ventricular mass index; UFV: ultrafiltration volume; UV: urine volume.

^a^LVEDV >86 ml/m^2^.

^b^LVMI ≥115 g/m^2^ for males and ≥95 g/m^2^ for females.

### Survival analysis

**Figure 2:**
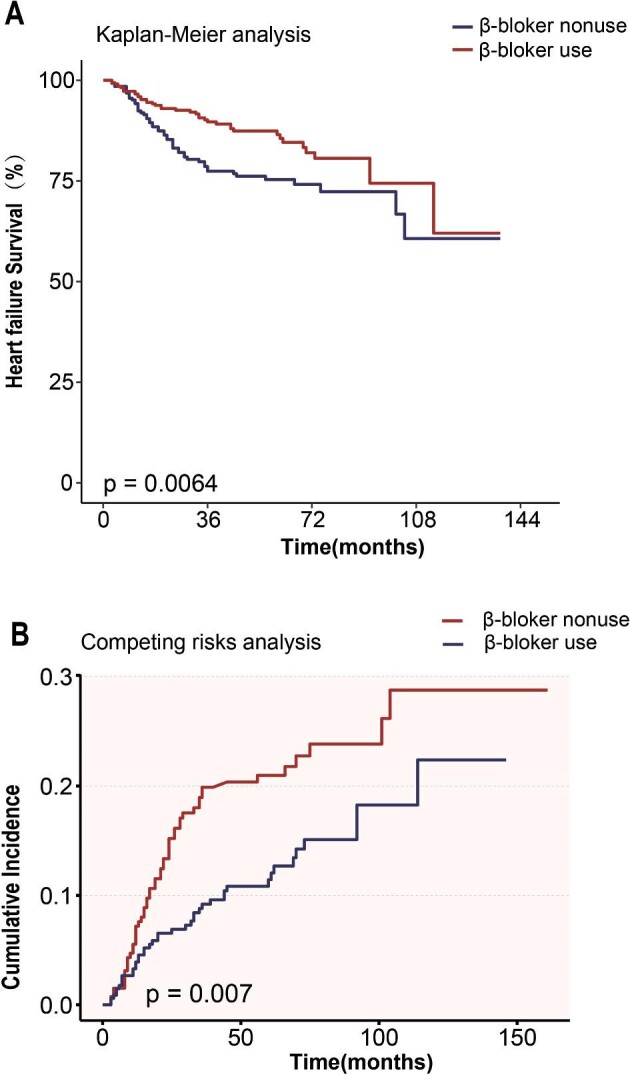
(**A**) Survival curves for Kaplan–Meier analysis of the association between β-blocker use and HF mortality (log-rank test). (**B**) Cumulative incident function curve for competing risks analysis of the association between β-blocker use and HF mortality (Gray's test).

Figure 2A depicts the Kaplan–Meier survival estimates for heart failure mortality, stratified by the presence or absence of β-blocker usage. Notably, the group receiving β-blockers exhibited a higher survival rate in relation to heart failure. The cumulative survival probabilities for β-blocker users versus non-users were 89.7% versus 77.4% at 3 years, and 86.5% versus 75.3% at 5 years. The cumulative incidence function curve in Fig. 2B shows that, even after adjusting for competing risk events, patients in the β-blocker group had a significantly lower cumulative risk of heart failure-related mortality compared to patients not on β-blockers (*P* = 0.007).

### Analysis of factors associated with heart failure mortality

Our univariate Cox regression analysis, presented in Table 3, demonstrated significant associations between various factors and the incidence of heart failure-related deaths. β-blocker use, urine output and albumin level showed a negative correlation with the occurrence of heart failure-related deaths. Conversely, age, history of heart failure, diabetes, coronary heart disease, smoking, and CCI displayed a positive correlation.

**Table 3:** Cox univariate analysis of factors associated with heart failure mortality and all-cause mortality.

	HF motality		All-cause mortality	
Variable	HR (95%CI)	*P*	HR (95%CI)	*P*
β-blocker	0.55 (0.37, 0.83)	0.005	0.72 (0.54, 0.95)	0.019
ACEI or ARB	1.15 (0.77, 1.74)	0.494	1.25 (0.94, 1.65)	0.122
Antiplatelet	4.23 (2.81, 6.37)	< 0.001	1.19 (0.87, 1.65)	0.280
DM	5.44 (3.61, 8.23)	< 0.001	2.67 (2.01, 3.57)	< 0.001
HBP	5.27 (3.10, 8.96)	< 0.001	2.05 (1.53, 2.75)	< 0.001
CHD	7.53 (4.89, 11.6)	< 0.001	3.07 (2.12, 4.47)	< 0.001
HF history	15.62 (9.72, 25.11)	< 0.001	3.31 (2.48, 4.42)	< 0.001
Smoking	2.26 (1.46, 3.48)	< 0.001	1.85 (1.35, 2.52)	< 0.001
CCI (scores)	1.10 (1.08, 1.12)	< 0.001	1.08 (1.06, 1.1)	< 0.001
Age (years)	1.08 (1.07, 1.1)	< 0.001	1.06 (1.05, 1.07)	< 0.001
BMI (kg/m^2^)	1.03 (0.98, 1.08)	0.321	1.00 (0.97, 1.04)	0.812
UV (ml)^a^	0.98 (0.96, 1.00)	0.048	0.98 (0.97, 1.00)	0.032
Hb (g/L)^b^	0.92 (0.83, 1.02)	0.114	0.99 (0.98, 0.99)	< 0.001
ALB (g/L)	0.97 (0.94, 0.99)	0.031	0.96 (0.93, 0.99)	0.003
Ca × P (mg**^2^**/dl**^2^**)	0.99 (0.97, 1.00)	0.023	0.91 (0.85, 0.97)	0.002
PTH (pmol/L)	0.99 (0.98, 1.00)	0.134	0.99(0.99, 1.00)	0.001

ACEI: Angiotensin-Converting Enzyme Inhibitor; ARB: Angiotensin II Receptor Blocker; DM: Diabetes Mellitus; HBP: High blood pressure; CHD: Coronary heart disease; CCI: Charlson comorbidities index; BMI: Body mass index; UV: Urine volume; Hb: Hemoglobin; ALB: Albumin; Ca×P: Calcium-phosphorus product; PTH: Parathyroid Hormone. Continuous variables, unless otherwise noted, values represent hazard ratio per unit increase.

^a^Urine volume as a continuous variable per 100 ml increase.

^b^Hemoglobin as a continuous variable per 10 g/L increase.

### Association between β-blocker use and outcome

The main results of the analyses of the effect of β-blocker use on outcomes, as well as adjustment for associated factors, are shown in Table 4. Even after these adjustments, the usage of β-blockers was associated with lower HF-related mortality. The hazard ratios (HRs) along with their corresponding 95% confidence intervals (CIs) obtained from the two adjusted models were as follows: HR = 0.55 (95% CI: 0.35–0.87, *P* = 0.010); HR = 0.57 (95% CI: 0.36–0.89, *P* = 0.013), respectively. After propensity score matching, Cox regression showed a significantly lower hazard ratio (HR = 0.61, 95% CI: 0.38–0.97, *P* = 0.038). With inverse probability of weighting, the HR remained significantly lower (HR = 0.64, 95% CI: 0.43–0.97, *P* = 0.016). These results consistently indicate a statistically lower risk of HF-related mortality associated with β-blockers use. Baseline data before and after propensity score matching, ROC curves, and SMD plots are presented in the supplementary data (Table S1, Fig. S1, and Fig. S2, see online supplementary material) for a detailed assessment of propensity score model performance and covariate balance after matching. Furthermore, even when accounting for competing risk events, β-blocker use remained associated with a significant reduction in HF-related mortality. Table 4 presents HRs from the two adjusted models: HR = 0.57 (95% CI: 0.38–0.85, *P* = 0.006) and HR = 0.58 (95% CI: 0.38–0.86, *P* = 0.008), respectively. However, it is worth noting that the association between β-blockers and all-cause mortality did not demonstrate statistical significance.

**Table 4:** Association between β-blocker use and the outcome in the multivariable anaylsis, prospensity-score analyses, and competing-risk analysis.

Analysis	HF mortality	*P*	All-cause mortality	*P*
No.of events/no.of patients at risk(%)				
No β-blockers use	53(19.9%)		101 (37.8%)	
β-blockers use	41(12.0%)		99 (29.0%)	
Multivariate analysis (95% CI)				
Crude	0.55 (0.37, 0.83)	0.005	0.72 (0.54, 0.95)	0.019
Model 1	0.55 (0.35, 0.87)	0.010	0.87 (0.65, 1.17)	0.352
Model 2	0.57 (0.36, 0.89)	0.013	0.88 (0.65, 1.18)	0.390
with PSM^a^	0.61 (0.38, 0.97)	0.038	0.88 (0.65, 1.20)	0.420
with IPTW^b^	0.64 (0.43, 0.97)	0.016	0.87 (0.66, 1.14)	0.318
Competing-risk analysis (95% CI)				
Model 1	0.57 (0.38, 0.85)	0.006		
Model 2	0.58 (0.38, 0.86)	0.008		

Model 1: Adjusted for age, diabetes, blood pressure, previous history of HF, coronary heart disease, CCI, urine volume, albumin, smoking, antiplatelet drug use.

Model 2: Adjusted for the variables in model 1 plus hemoglobin, ACEI/ARB use, calcium-phosphorus product.

^a^Shown is the hazard ratio from the multivariable Cox proportional-hazards model with the same covariates with matching according to the propensity score. The analysis included 438 patients (219 who received β-blockers and 219 who did not.)

^b^Shown is the hazard ratio from the multivariable Cox proportional-hazards model with the same covariates with inverse probability weighting according to the propensity score.

### Subgroup analysis

**Figure 3:**
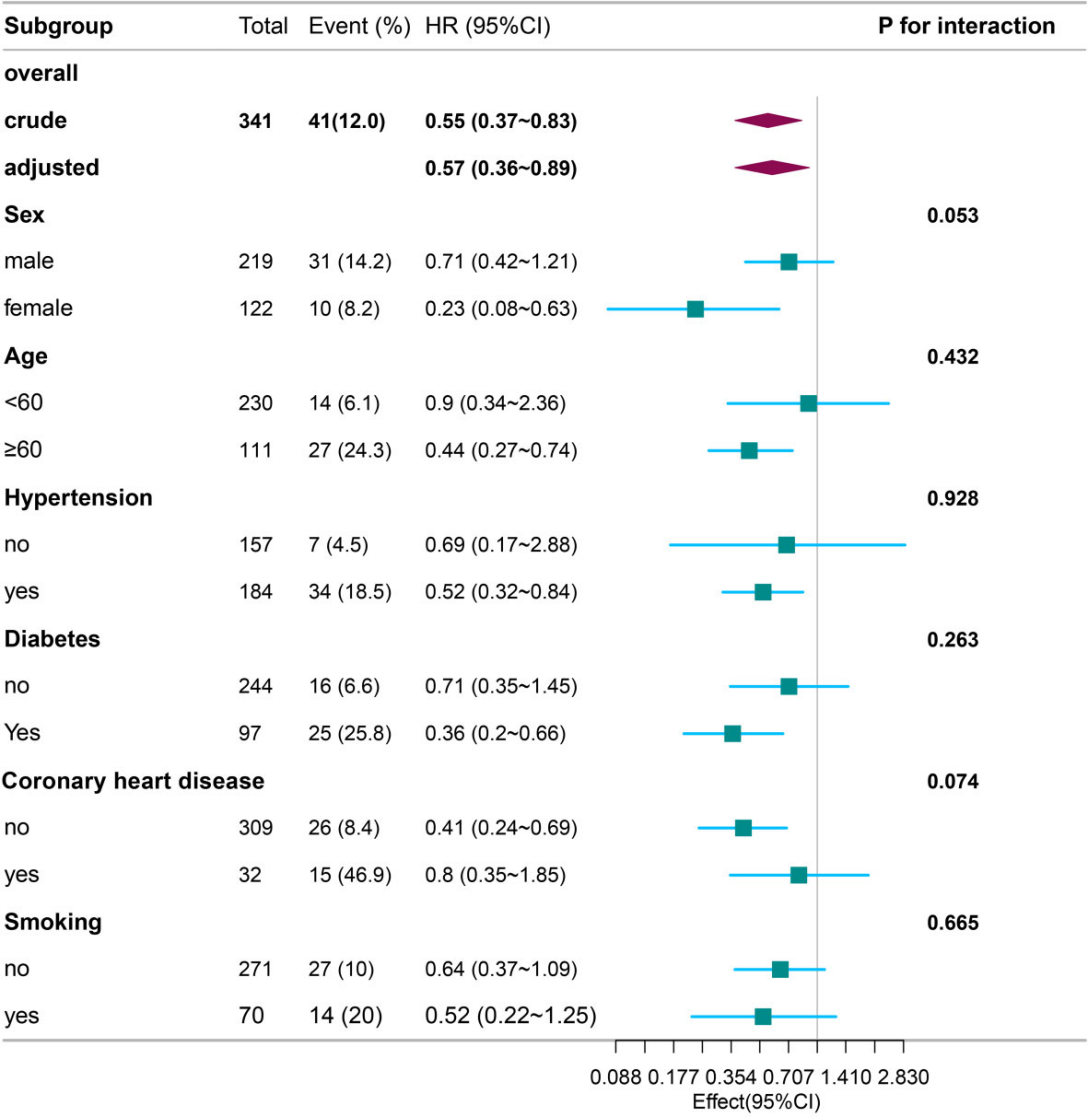
Forest plot with subgroup analyses of β-blocker and heart failure mortality.

Subgroup analyses were conducted to explore whether there were potential subgroup differences in the outcome of β-blocker utilization and mortality related to HF. Stratification variables encompassed age, sex, diabetes, history of coronary heart disease, smoking, and hypertension. The findings of these subgroup analyses, including any potential interactions, are presented in Fig. 3. Notably, the results of β-blocker use analysed in the aforementioned subgroups remained consistent with the overall results, with no significant interactions.

## DISCUSSION

To the best of our knowledge, our study is the first observational study focusing on the effects of β-blockers on mortality outcomes in heart failure in peritoneal dialysis population. This retrospective study has the following findings: (i) β-blockers are associated with a lower risk of heart failure-related mortality among patients undergoing PD; (ii) HFpEF is highly prevalent in the PD population.

Previous studies have demonstrated the beneficial effects of β-blocker therapy in patients with HFrEF at all stages of CKD population, including those on hemodialysis [28]. In fact, despite limited studies investigating the cardiovascular protective effects in the PD population, β-blockers are still commonly prescribed to these patients to manage blood pressure, atrial fibrillation, and coronary heart disease. However, cautious consideration is warranted by clinicians due to previous study findings suggesting potential adverse effects, including the development of sclerosing encapsulating peritonitis and a decrease in peritoneal ultrafiltration [30–32]. Ultrafiltration volume control plays a pivotal role in predicting outcomes in chronic PD patients, as salt and fluid removal are integral components of cardiovascular management in this population. It is noteworthy that fluid removal strategies heavily rely on peritoneal ultrafiltration. Nevertheless, in our evaluation of patients who experienced HF events in this study, no statistically significant differences in ultrafiltration and urine output were apparent between the groups using β-blockers and those not utilizing them. Additionally, there was no observed occurrence of encapsulated peritonitis in the population of β-blocker users. Sensitivity analyses of different dimensions used in our study all support the association of β-blockers with a lower risk of death from HF.

Mechanistically, the beneficial effects of β-blockers in patients with heart failure are well-established. These medications reduce sympathetic outflow, resulting in decreased heart rate, blood pressure, and myocardial oxygen demand. This may alleviate the strain on the cardiovascular system and improve cardiac function in patients. Additionally, patients on PD may have distinct pathophysiological mechanisms and clinical characteristics than those with advanced CKD. These include malnutrition [33], continuous peritoneal glucose exposure leading to peritoneal stimulation [34], dyspepsia, and gastrointestinal abnormalities that stimulate the production of inflammatory factors by intestinal bacteria, thereby perpetuating a persistent inflammatory state [35, 36]. In contrast, β-blockers possess anti-inflammatory and anti-fibrotic properties [37, 38], potentially improving the inflammatory state of the body and mitigating the adverse effects of peritoneal membrane dysfunction and cardiovascular remodeling.

Furthermore, we noted a 43.4% prevalence of heart failure episodes among peritoneal dialysis patients, aligning with the reported findings of a 2011 study on peritoneal dialysis (87 out of 220 patients), which documented a similar prevalence of 40.9% [5]. Although follow-up data on LVEF were missing for 29 patients with HF, LVEF <50% was observed in only 25 of 264 patients with HF in our study, suggesting that the proportion of HFrEF is lower than that of HFpEF. In 2013, a study was conducted that evaluated HF in 220 cases of PD and showed that 86 cases developed HF and the percentage of HFpEF (54.7%) was also greater than that of HFrEF (45.3%) [39]. HFpEF, characterized by concentric myocardial hypertrophy, diastolic dysfunction, and LVEF ≥50%, is estimated to affect one‐third of all HF patients and is more prevalent in PD patients [39, 40]. HFpEF usually results from a combination of risk factors and comorbidities, with renal failure, hypertension, diabetes mellitus, volume overload, and anemia being important contributors and common comorbidities in PD patients. In addition, PD patients have chronically elevated intra-abdominal pressure, which affects inferior vena cava reflux and right ventricular filling and has a relatively minor effect on left ventricular systolic function. These may partly explain the high proportion of HFpEF in the PD population.

Consistent with previous studies conducted in the CKD and hemodialysis population, our study contributes to the existing literature by providing evidence on the potential cardiovascular benefit of β-blocker therapy in PD patients. Nevertheless, there are several limitations to our study that should be acknowledged. Firstly, despite our diligent efforts to minimize confounders and partially mitigate indication bias through propensity score inverse probability treatment weighting, it is essential to acknowledge that the observational data utilized in this study remained susceptible to selection and treatment biases, which could not be entirely eradicated. The prescription of β-blockers in ESRD patients undergoing PD was not randomized, and clinicians may have reservations about prescribing β-blockers due to concerns regarding volume overload, heart rate fluctuations, or exacerbation of heart failure symptoms. Therefore, a causal relationship with HF-related mortality cannot be established and stronger evidence from prospective studies or randomized controlled trials would provide more robust evidence in this regard. Secondly, although we adjusted for several confounders, the potential existence of unobserved latent factors may have led to an overestimation of the observed associations. Moreover, our study lacked the capability to assess adverse drug reactions or modifications in drug dosage post-study initiation, introducing a potential limitation in the comprehensiveness of our findings. Thirdly, our study was conducted at a single center with a small sample size, which may limit the generalizability of our findings to other settings. It is important to replicate these investigations in larger and more diverse cohorts to ensure external validity.

## CONCLUSION

Our study provides valuable insights into the prognostic significance of β-blocker therapy in PD with HF. We observed that β-blocker utilization was associated with a lower risk of heart failure mortality, independent of comorbidities such as cardiovascular disease or hypertension. These findings underscore the potential benefits of β-blockers in the PD population and advocate for further randomized clinical trials to validate and expand upon these observations. Ultimately, optimizing therapeutic strategies for PD patients can contribute to improved clinical outcomes and enhance the quality of life for this vulnerable patient population.

## Supplementary Material

sfae022_Supplemental_File

## Data Availability

The data underlying this article will be shared on reasonable request to the corresponding author.
