# Supplementary material for: Association between beta-blocker utilization and heart failure mortality in the peritoneal dialysis population: a cohort study
Source: Clin Kidney J. 2024 Feb 9;17(3):sfae022. doi: 10.1093/ckj/sfae022 (PMC10913941; doi:10.1093/ckj/sfae022)
Supplement: sfae022_Supplemental_File [file sfae022_supplemental_file.pptx]

## Slide 1
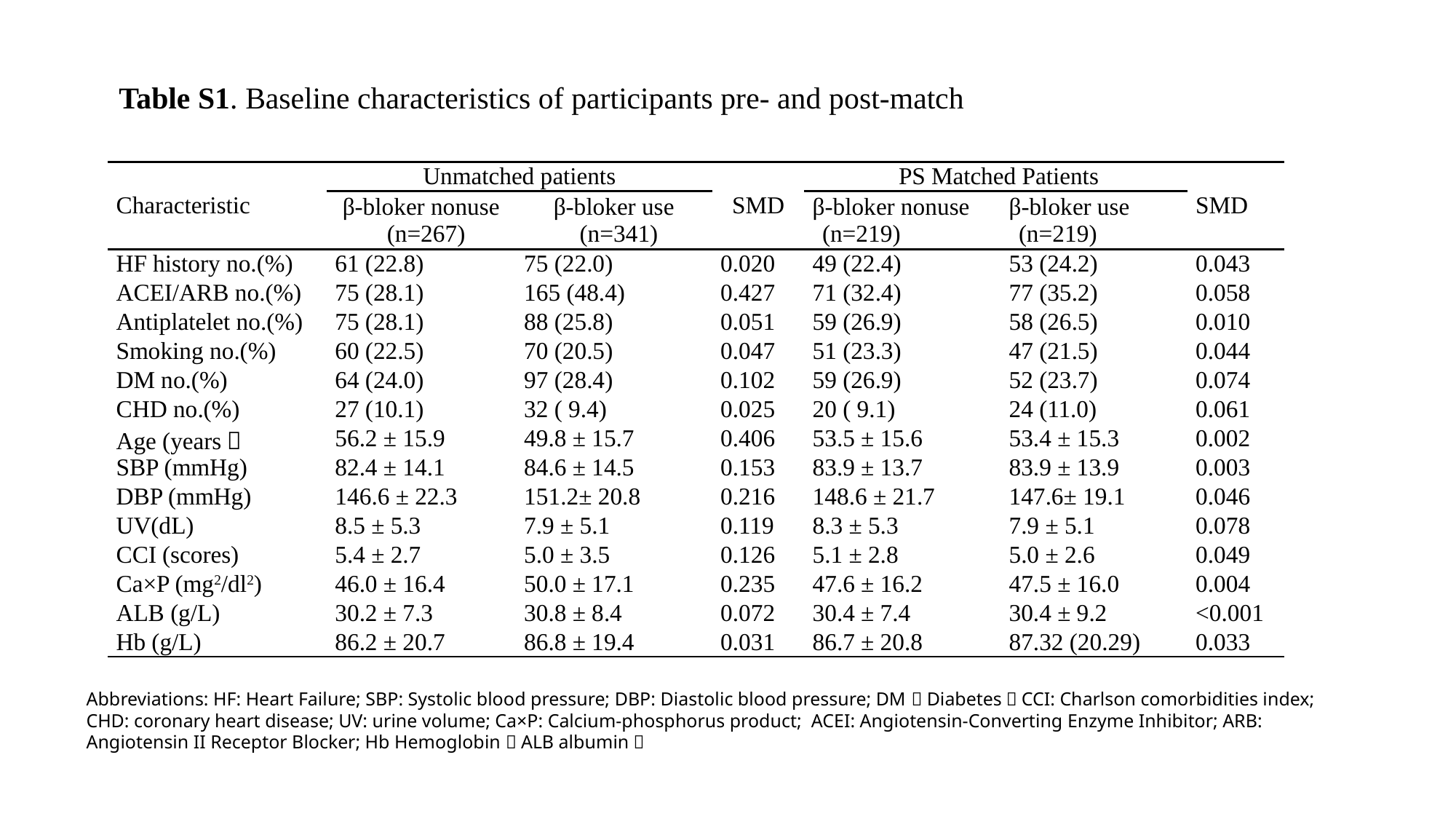

Table S1. Baseline characteristics of participants pre- and post-match
| Characteristic | Unmatched patients | | SMD | PS Matched Patients | | SMD |
| --- | --- | --- | --- | --- | --- | --- |
| | β-bloker nonuse (n=267) | β-bloker use (n=341) | | β-bloker nonuse (n=219) | β-bloker use (n=219) | |
| HF history no.(%) | 61 (22.8) | 75 (22.0) | 0.020 | 49 (22.4) | 53 (24.2) | 0.043 |
| ACEI/ARB no.(%) | 75 (28.1) | 165 (48.4) | 0.427 | 71 (32.4) | 77 (35.2) | 0.058 |
| Antiplatelet no.(%) | 75 (28.1) | 88 (25.8) | 0.051 | 59 (26.9) | 58 (26.5) | 0.010 |
| Smoking no.(%) | 60 (22.5) | 70 (20.5) | 0.047 | 51 (23.3) | 47 (21.5) | 0.044 |
| DM no.(%) | 64 (24.0) | 97 (28.4) | 0.102 | 59 (26.9) | 52 (23.7) | 0.074 |
| CHD no.(%) | 27 (10.1) | 32 ( 9.4) | 0.025 | 20 ( 9.1) | 24 (11.0) | 0.061 |
| Age (years） | 56.2 ± 15.9 | 49.8 ± 15.7 | 0.406 | 53.5 ± 15.6 | 53.4 ± 15.3 | 0.002 |
| SBP (mmHg) | 82.4 ± 14.1 | 84.6 ± 14.5 | 0.153 | 83.9 ± 13.7 | 83.9 ± 13.9 | 0.003 |
| DBP (mmHg) | 146.6 ± 22.3 | 151.2± 20.8 | 0.216 | 148.6 ± 21.7 | 147.6± 19.1 | 0.046 |
| UV(dL) | 8.5 ± 5.3 | 7.9 ± 5.1 | 0.119 | 8.3 ± 5.3 | 7.9 ± 5.1 | 0.078 |
| CCI (scores) | 5.4 ± 2.7 | 5.0 ± 3.5 | 0.126 | 5.1 ± 2.8 | 5.0 ± 2.6 | 0.049 |
| Ca×P (mg2/dl2) | 46.0 ± 16.4 | 50.0 ± 17.1 | 0.235 | 47.6 ± 16.2 | 47.5 ± 16.0 | 0.004 |
| ALB (g/L) | 30.2 ± 7.3 | 30.8 ± 8.4 | 0.072 | 30.4 ± 7.4 | 30.4 ± 9.2 | <0.001 |
| Hb (g/L) | 86.2 ± 20.7 | 86.8 ± 19.4 | 0.031 | 86.7 ± 20.8 | 87.32 (20.29) | 0.033 |
Abbreviations: HF: Heart Failure; SBP: Systolic blood pressure; DBP: Diastolic blood pressure; DM：Diabetes；CCI: Charlson comorbidities index; CHD: coronary heart disease; UV: urine volume; Ca×P: Calcium-phosphorus product; ACEI: Angiotensin-Converting Enzyme Inhibitor; ARB: Angiotensin II Receptor Blocker; Hb Hemoglobin；ALB albumin；

## Slide 2
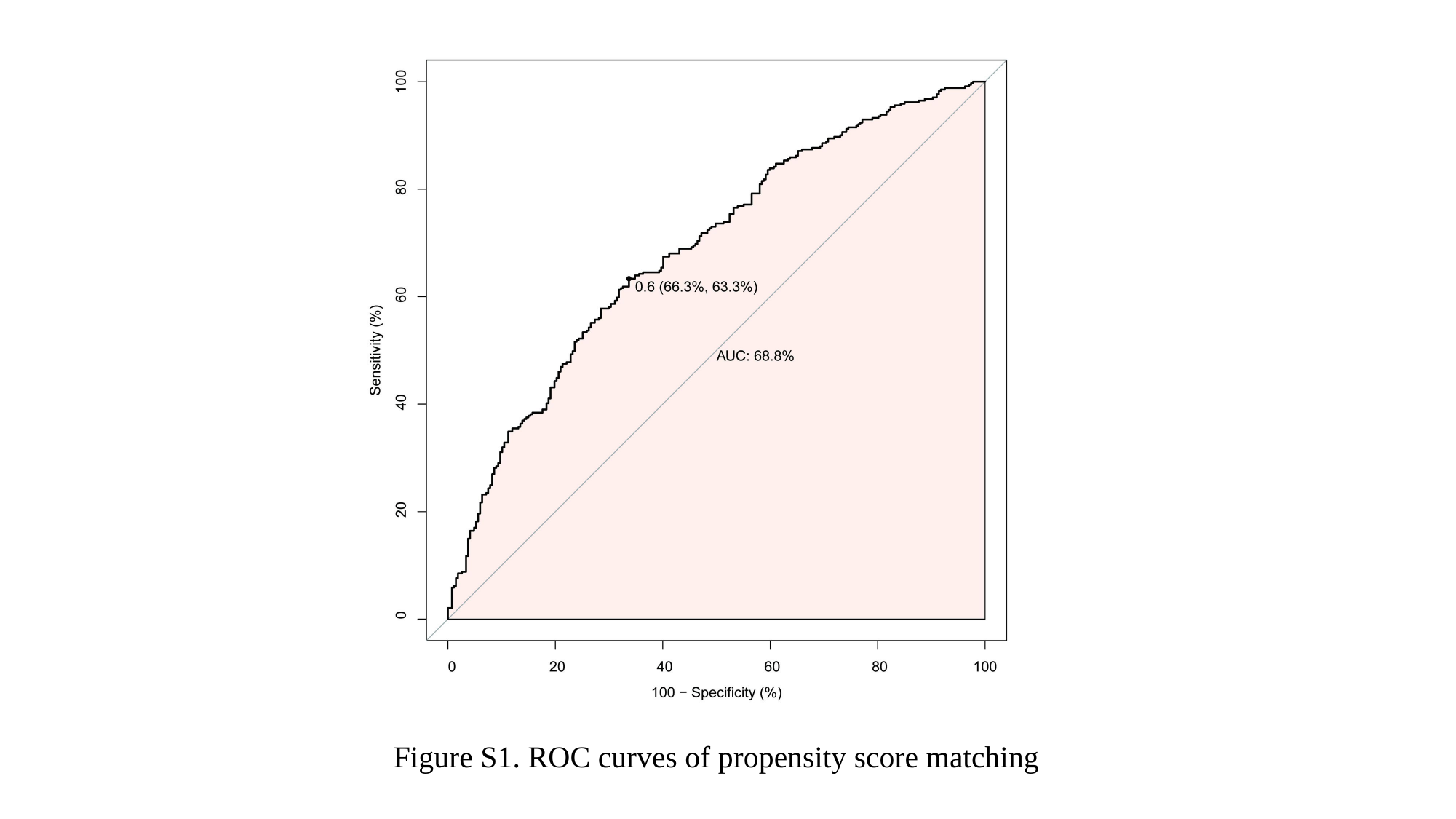

Figure S1. ROC curves of propensity score matching

## Slide 3
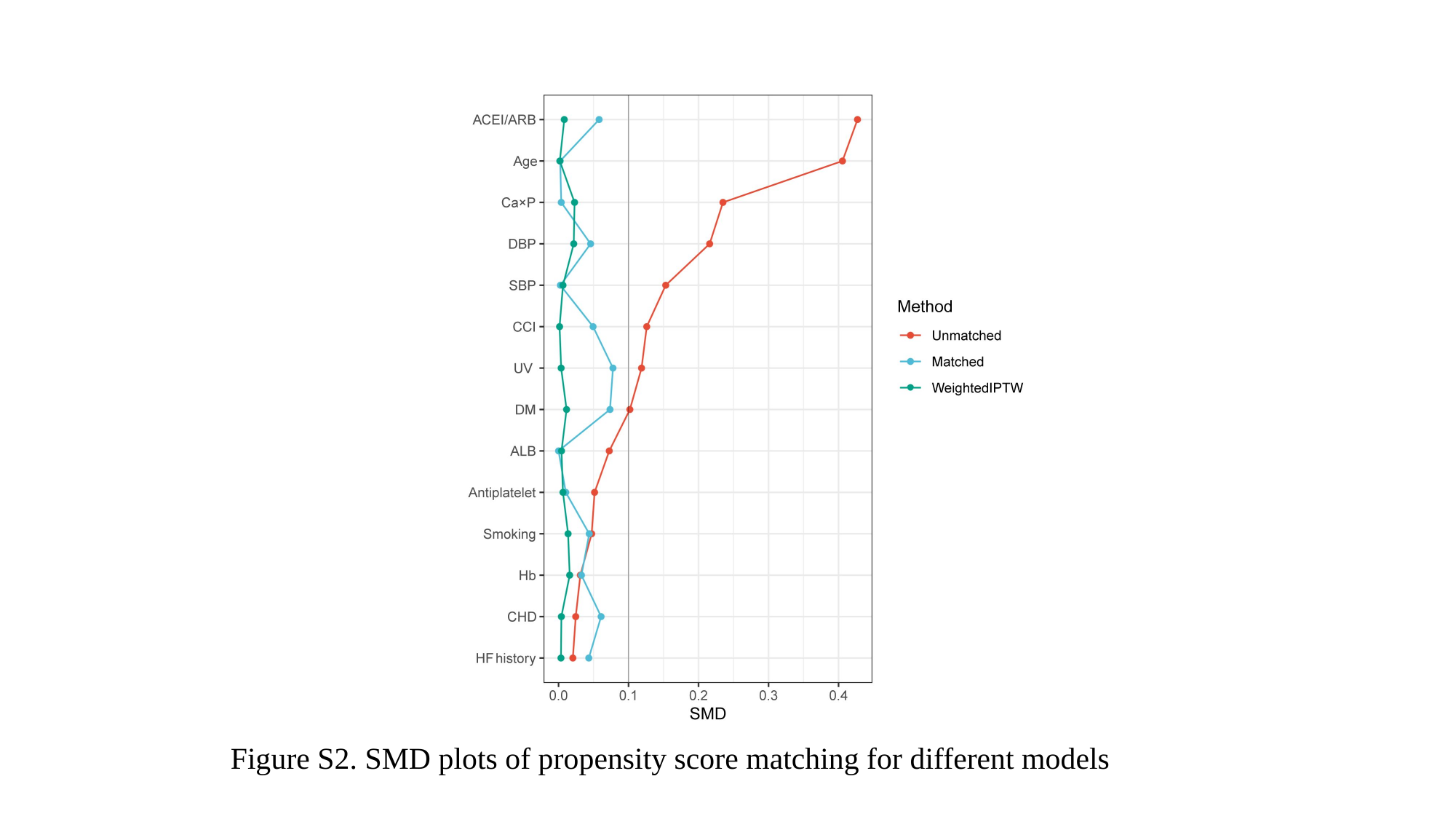

Figure S2. SMD plots of propensity score matching for different models
